# Facile phase transfer of gold nanorods and nanospheres stabilized with block copolymers

**DOI:** 10.3762/bjnano.9.58

**Published:** 2018-02-16

**Authors:** Yaroslav I Derikov, Georgiy A Shandryuk, Raisa V Talroze, Alexander A Ezhov, Yaroslav V Kudryavtsev

**Affiliations:** 1 Topchiev Institute of Petrochemical Synthesis, Russian Academy of Sciences, Leninsky Prosp. 29, 119991 Moscow, Russia; 2 Faculty of Physics, Lomonosov Moscow State University, Leninskie gory 1–2, 119991, Moscow Russia; 3 Frumkin Institute of Physical Chemistry and Electrochemistry, Russian Academy of Sciences, Leninsky Prosp. 31, 119071 Moscow, Russia

**Keywords:** Au nanorods, block copolymers, optical absorbance spectroscopy, phase transfer, seeded growth method

## Abstract

A fast route to transfer Au nanoparticles from aqueous to organic media is proposed based on the use of a high molecular mass diblock copolymer of styrene and 2-vinylpyridine for ligand exchange at the nanoparticle surface. The method enables the preparation of stable sols of Au nanorods with sizes of up to tens of nanometers or Au nanospheres in various organic solvents. By comparing the optical absorbance spectra of Au hydro- and organosols with the data of numerical simulations of the surface plasmon resonance, we find that nanoparticles do not aggregate and confirm the transmission electron microscopy data regarding their shape and size. The proposed approach can be effective in preparing hybrid composites without the use of strong thiol and amine surfactants.

## Introduction

The size effects that determine the functional characteristics of nanoparticles are no less important than their precise positioning in host matrices. Control over the size, shape and surface of nanoparticles is an effective tool that can be used in bottom up approaches for the fabrication of composite materials [1–2]. An optimal strategy of nanoparticle synthesis should account for their target application. For example, block copolymers doped with noble metal nanoparticles are considered as promising hybrid materials that combine the plasmonic, catalytic or other functionality of nanoparticles, their periodic spatial arrangement in the microphase separated copolymer domains and the general processability of polymer matrices [3–4]. Nanoparticles suitable for this purpose should be stable against aggregation, miscible with at least one of the copolymer blocks and should match the copolymer domain size and shape. The major part of the block copolymer phase diagram is occupied by cylinder and lamellar phases [5], which are intrinsically anisotropic. Their possible coupling with nanorods, as the simplest anisometric nanoparticles, is of emerging interest in recent experimental [6–8] and theoretical [9–12] research.

In the case of Au nanoparticles, which are the focus of the current study, a number of synthetic routes are available, with the seeded growth method being the most popular in terms of size and shape control [13]. Its current version [14–16] allows one to obtain Au nanorods with a high yield and desired aspect ratio ranging from 1.5 to 5. The water-soluble nanoparticles synthesized in this way require organophilization before introducing them into a polymer matrix, except for when the host polymer is hydrophilic. The so-called phase transfer from polar to non-polar media can be carried out in a number of ways [17]. A typical procedure includes intensive mixing of a hydrosol of citrate-stabilized Au nanospheres or cetyl trimethylammonium bromide-stabilized Au nanorods with a solution of thiol- [18] or amine-terminated [19] surfactants in organic solvent. With a strong affinity to gold, thiols and amines replace nanoparticle shells via ligand exchange, while the tails of these molecules provide miscibility in non-polar media. In some cases, for instance, when an organic solvent is poorly miscible with water, more complex schemes involving phase transfer catalysts [20], electrostatics [21–22] or sonication [23] are implemented. Large (>10 nm) nanoparticles are prone to aggregation in organic solvents due to van der Waals interactions, so their stabilization requires the use of bulky surfactant molecules [24–26] or polymers with a strongly binding groups via grafting-to [27–28], grafting-from [4] and grafting-around [29] approaches, hyperbranched polymers [30] or polymers with a significant number of interacting pendant groups that provide nanoparticle “wrapping” [31–33].

Block copolymers can also be used for the stabilization of Au nanoparticles via grafting-to [34–35], grafting-from [36] and wrapping [29,37] strategies. Since nanoparticles can be driven by laser light [36], they enhance sensitivity of amphiphilic dispersions to external stimuli. Block copolymers are effective for the phase transfer of spherical Au nanoparticles up to 30 nm in diameter from aqueous to organic phases [29,35–36]. The tendency for aggregation is increased when the block interacting with the nanoparticle surface is shortened.

In this study, we propose a facile route to organophilization of Au nanoparticles using a high molecular mass diblock copolymer of styrene and 2-vinylpyridine. Our method provides fast phase transfer of nanorods of different sizes and aspect ratios and their redispersion in various organic solvents. The same copolymer can be further subjected to microphase separation so that no third component compatibilizer in the hybrid composite material is needed.

## Results and Discussion

### Au nanorod synthesis and phase transfer

We synthesized Au nanorods by the seeded growth method [14–16], as described in the Experimental section. We also tried the seedless technique proposed in [16], but obtained a mixture of rod-like and (prevailing) spherical particles. Though it appeared possible to increase the content of nanorods using fractional centrifugation, we chose to adhere to the approved two-stage technique.

We transferred Au nanorods to organic media via 5 min of mixing of their hydrosol with a solution of PS-b-P2VP diblock copolymer in tetrahydrofuran. After drying, redissolution in tetrahydrofuran, precipitation by ethanol, centrifugation, and again drying, nanorods were dispersible in tetrahydrofuran, chloroform, methylene chloride, toluene and benzene. The phase transfer procedure is fully described in the Experimental section.

**Figure 1:**
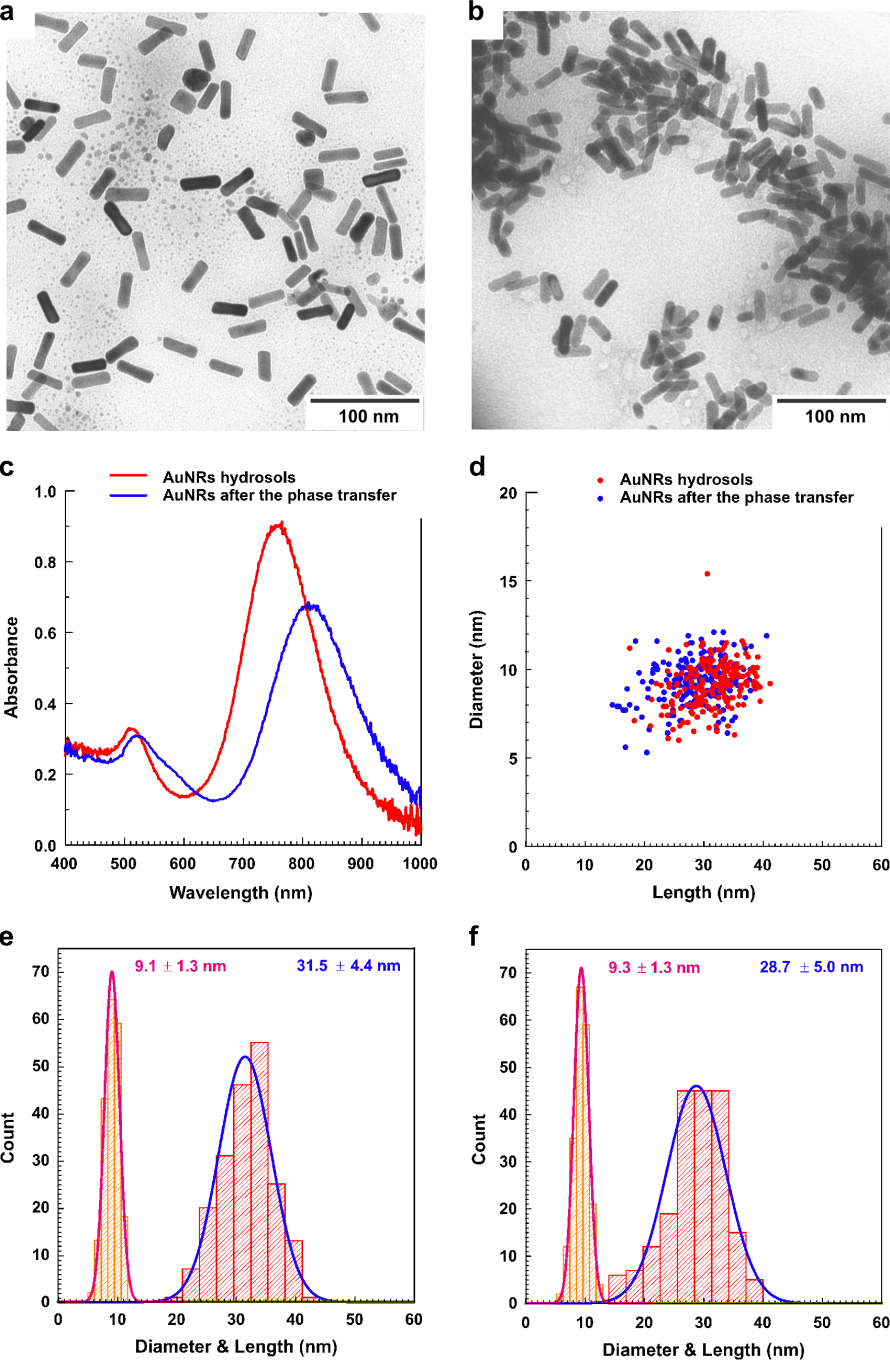
Comparison of the morphological and optical characteristics of the initial Au hydrosol and the polymer-coated Au nanorod sol in benzene. TEM images of Au nanorods (a) before and (b) after phase transfer, (c) comparison of optical absorbance spectra in water and benzene and (d) 2D diagram of measured lengths and diameters and Au nanorods distributions over length and diameter (e) before and (f) after the phase transfer. The curves and numbers define the approximating normal distributions.

The morphology and optical characteristics of the initial Au nanorod hydrosol and of the polymer-coated Au sol dried from benzene are shown in Figure 1. The results of the statistical analysis of the nanorod geometry from a series of transmission electron microscopy (TEM) images are listed in Table 1. The small values of the Pearson coefficients in Table 1 and the shapes of the scatter plots in Figure 1 indicate the absence of correlations between the length and diameter of nanorods.

**Table 1:** Nanorod size parameters from TEM data.

Sample	NP number	Parameter	Mean/Median	Standard deviation	Min/Max	Pearson correlation coefficient

AuNR hydrosol	200	length	31.5/31.9 nm	4.4 nm	17.5/41.2 nm	0.07
		diameter	9.1/9.2 nm	1.3 nm	6.0/15.4 nm	
		aspect ratio	3.5/3.5	0.6	1.6/5.6	–
AuNRs after the phase transfer	200	length	28.7/29.2 nm	5.0 nm	14.6/40.6 nm	0.08
		diameter	9.3/9.3 nm	1.3 nm	5.3/12.1 nm	
		aspect ratio	3.1/3.1	0.6	1.6/5.3	–

The length (31.5 ± 4.4 nm) and diameter (9.1 ± 1.3 nm) of phase transferred nanorods are ≈10% less than the values measured by TEM for the initial hydrosol (28.7 ± 5.0 nm and 9.3 ± 1.3 nm), whereas the mean aspect ratio is kept nearly constant (decreased from 3.5 to 3.1). A small decrease in the nanorod mean size can be explained by the weak selectivity of the phase transfer method, if we admit that longer Au nanorods are transferred slightly worse than shorter ones. Nevertheless, the proposed method of phase transfer is effective even for large particles of tens of nanometer size. Following the literature studies [37–38], we can speculate regarding the preferable interaction of gold with pyridine groups (P2VP-coated Au nanoparticles can be synthesized from HAuCl_4_ in tetrahydrofuran [39]) that leads to wrapping nanorods with P2VP copolymer blocks, while PS blocks form coronas preventing nanorod aggregation.

### Modeling the extinction spectra of Au nanorods

Two bands in the optical absorbance spectra (Figure 1b) of the Au nanorods are attributed to the transverse (TE) and longitudinal (TM) plasmon modes, respectively [40]. The position of the former band, which is the only one observable for Au nanospheres, only weakly depends on the nanoparticle size and geometry, while the latter band is considerably shifted toward the near-infrared region with increasing nanorod aspect ratio [41].

We simulated the interaction of light with spherical and rod-like (cylindrical) Au nanoparticles in various media in order to model their extinction spectra. On this basis, we employed Lumerical Solutions software based on solving Maxwell’s equations with the finite-difference time-domain numerical technique. The simulations were carried out for a linearly polarized light wave with the electric field set parallel (TM) or perpendicular (TE) to the cylinder long axis. The obtained spectra were averaged for all possible nanorod orientations. The aim of the simulations was twofold: (i) to confirm that, both in water and in an organic medium, Au nanorods exist as separate particles rather than their aggregates and (ii) to obtain information regarding the surface layer that stabilizes the nanoparticles before and after the phase transfer.

**Figure 2:**
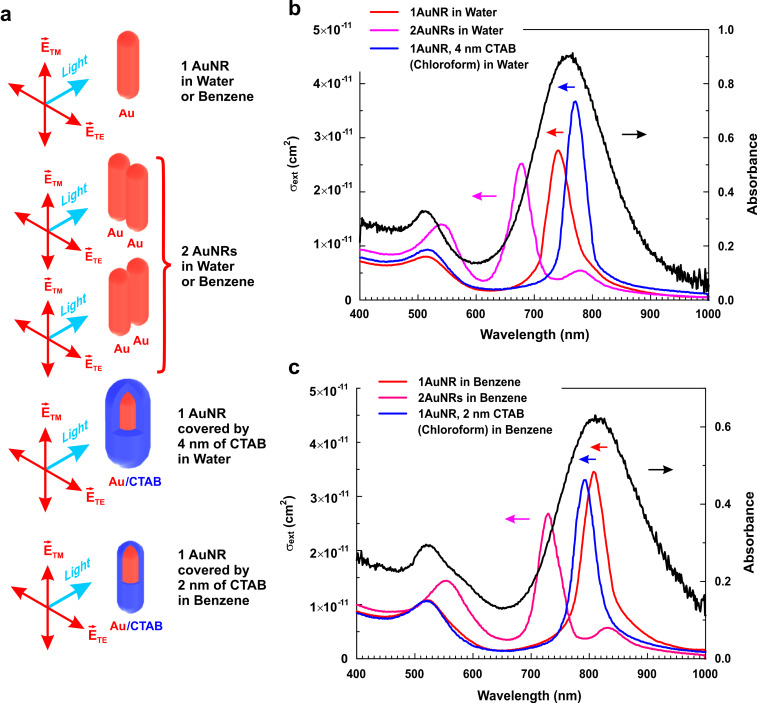
(a) Diagrams illustrating the geometry used in our simulations. (b) Comparison of the calculated extinction cross sections for a bare single Au nanorod (1AuNR), a dimer of two Au nanorods (2AuNRs) and a single Au nanorod coated with 4 nm of CTAB (1AuNR, 4 nm CTAB) in water with the experimental optical absorbance spectrum shown in Figure 1. (c) Comparison of the calculated extinction cross sections for a bare single Au nanorod (1AuNR), a dimer of two Au nanorods (2AuNRs) and a single Au nanorod coated by 2 nm of CTAB (1AuNR, 2 nm CTAB) in benzene with the experimental optical absorbance spectrum shown in Figure 1 (after the phase transfer).

The first task stemmed from our TEM observations that demonstrated the possibility of nanorod side-by-side stacking (see Figure 1a). Such a mutual arrangement of nanorods could arise either during solvent evaporation or earlier in a sol state. In order to shed light on the behavior of Au nanorods in hydrosols and organosols, we modeled the extinction spectra of a single rounded cylinder and of a simplest possible aggregate containing two such cylinders positioned in parallel and spaced 1 nm apart. Based on the statistical analysis of the TEM data, the model nanorods were chosen to be of 31.5 nm length and 9.1 nm diameter in water and of 28.7 nm length and 9.3 nm diameter in benzene. The literature data for the optical dispersion properties of gold [42], water [43] and benzene [44] were used. By comparing the modeled extinction cross sections with the experimental optical absorbance spectra in Figure 2, one can see that, both in water and benzene, the experimental data are much closer to the simulation results for a single nanorod rather than for a dimer. Thus, the simulations corroborate our estimates of the nanorod mean length and diameter by TEM and indicate that side-by-side stacking of Au nanorods and their aggregation in general are not typical for our system.

**Figure 3:**
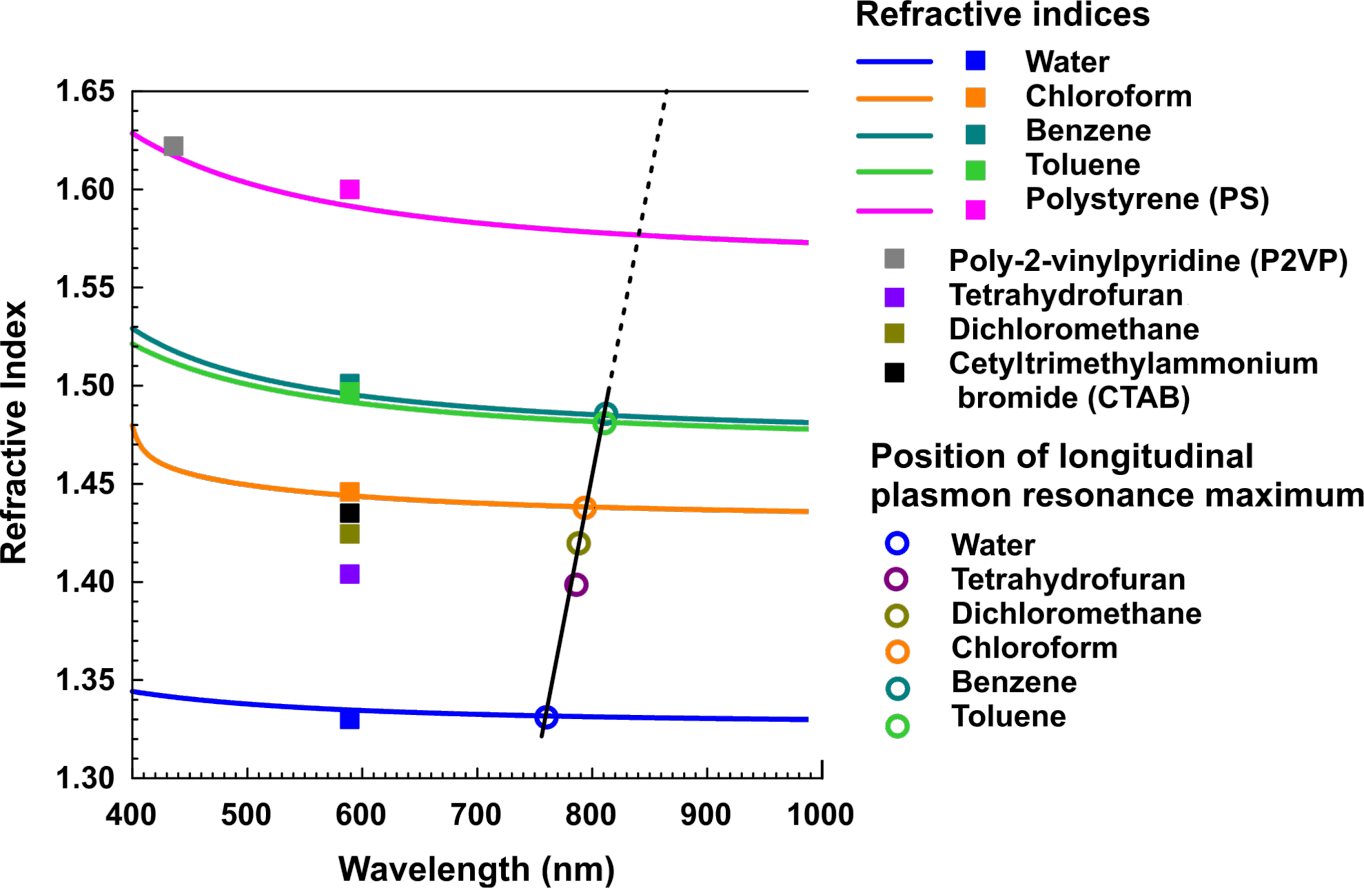
Refractive indices of the substances used in this study (excluding gold). Hollow circles indicate the spectral position of the longitudinal plasmon resonance of Au nanorods obtained for a 5 min seeding time. The data were taken from [43–44,48–52].

It is known [45–46] that the TM and TE plasmon bands for nanorods depend on the refractive index of a medium bordering the nanorod surface. The effect is more pronounced for the TM mode. As shown in [47], in hydrosols, CTAB forms a 4–5 nm bilayer at the surface of Au nanoparticles. The optical dispersion curves for the substances relevant to this study are presented in Figure 3. For the sodium D-line, the refractive index of CTAB equals 1.4350 [48], which is close to 1.4440 for chloroform [49], but markedly different from 1.3333 for water [43], 1.4956 for benzene [44], 1.4920 for toluene [44], 1.5915 for polystyrene [50] and 1.622 for poly(2-vinylpyridine) at 436 nm [51]. Thus, the presence of a CTAB bilayer at the Au nanorod surface should noticeably shift the maximum position of the TM plasmon resonance in water and benzene. This can be checked by simulations for a model in which a single nanorod is coated with a surface layer with the refractive index differing from that of a dispersion medium. In the absence of any data on the CTAB refractive index dispersion in the 400–1000 nm spectral range, we replaced it with the corresponding data for chloroform [49]. The simulations were carried out for an Au rounded cylinder with a 31.5 nm length and a 9.1 nm diameter coated with a continuous CTAB (chloroform) shell with a 4 nm thickness, placed in water. The resulting extinction cross section is shown in Figure 2b, along with the spectrum describing the model system without CTAB and with the experimental optical absorbance spectrum. Since the experimental band maximum is situated between the peaks of two model spectra, we can conclude that some CTAB is present at the nanorod surface but its quantity is not enough to form a uniform bilayer. Presumably, the bilayer is rarefied near the nanorod ends.

In nonpolar organosols, CTAB should form a ca. 2 nm monolayer at Au surfaces. For the simulations, we took a rounded Au nanorod with a 28.7 nm length and a 9.3 nm diameter coated with a continuous 2 nm shell, placed in benzene. The model spectrum is shown in Figure 2c. It is seen that its maximum is farther from the experimental value that the maximum of the model spectra for the bare Au nanorod. However, we should bear in mind that the experimental optical absorbance spectrum of Au nanorods in benzene was recorded after the phase transfer, which is described in detail in the next section. If we suppose that during the phase transfer CTAB is partially replaced with the PS-b-P2VP block copolymer, the presence of polystyrene blocks in the surface layer of nanorods can effectively increase the refractive index of that layer and bring it close to the refractive index of the dispersion medium, benzene. If so, the modeled extinction cross section for a bare nanorod would be close to the experimental absorption spectrum, as one can see from Figure 2c. In any case, we can conclude that Au nanorods in benzene are not coated with a continuous layer of CTAB.

### Nanorod behavior in organic solvents

**Figure 4:**
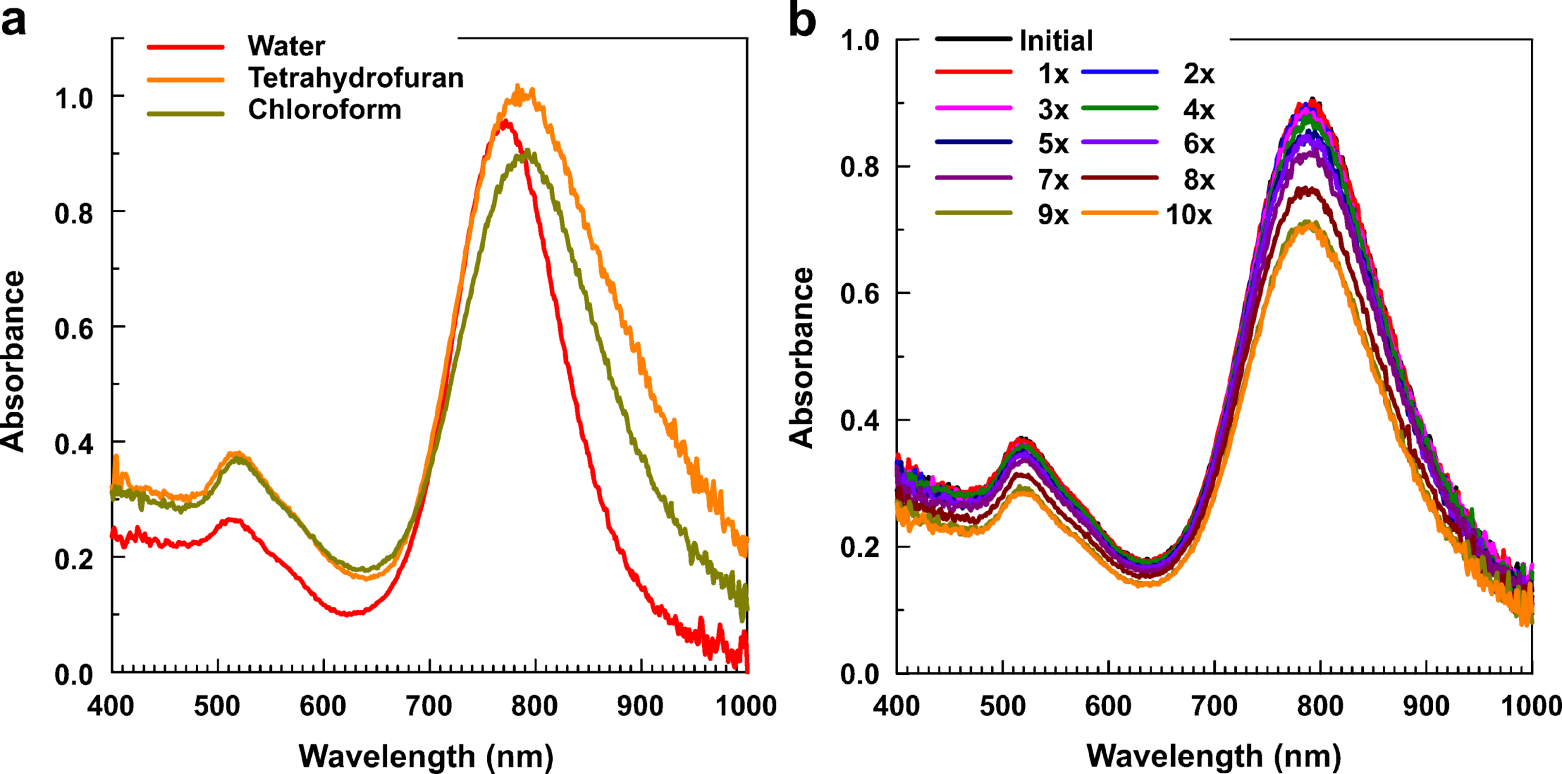
(a) Optical absorbance spectra of Au nanorods (water) before the phase transfer, (tetrahydrofuran) during the transfer and (chloroform) after the transfer. (b) The same spectra recorded during ten consecutive redistributions (from 1× to 10×) of the Au sol in chloroform.

The absorption spectra taken (in tetrahydrofuran) during the phase transfer of Au nanorods and (in chloroform) after the transfer look very similar. Their transverse and longitudinal bands are red-shifted from those bands for the initial Au hydrosol by ca. 10 and 20 nm, respectively (Figure 4a).

The polymer-coated Au nanorods reveal remarkable stability. As shown in Figure 4b, ten cycles of vacuum drying and redispersion of the sol in chloroform do not affect the plasmon band positions. About 20% decrease in the peak amplitude is comparable to the losses from the sample transfer to and from the spectrometer cuvette after ten cycles.

**Figure 5:**
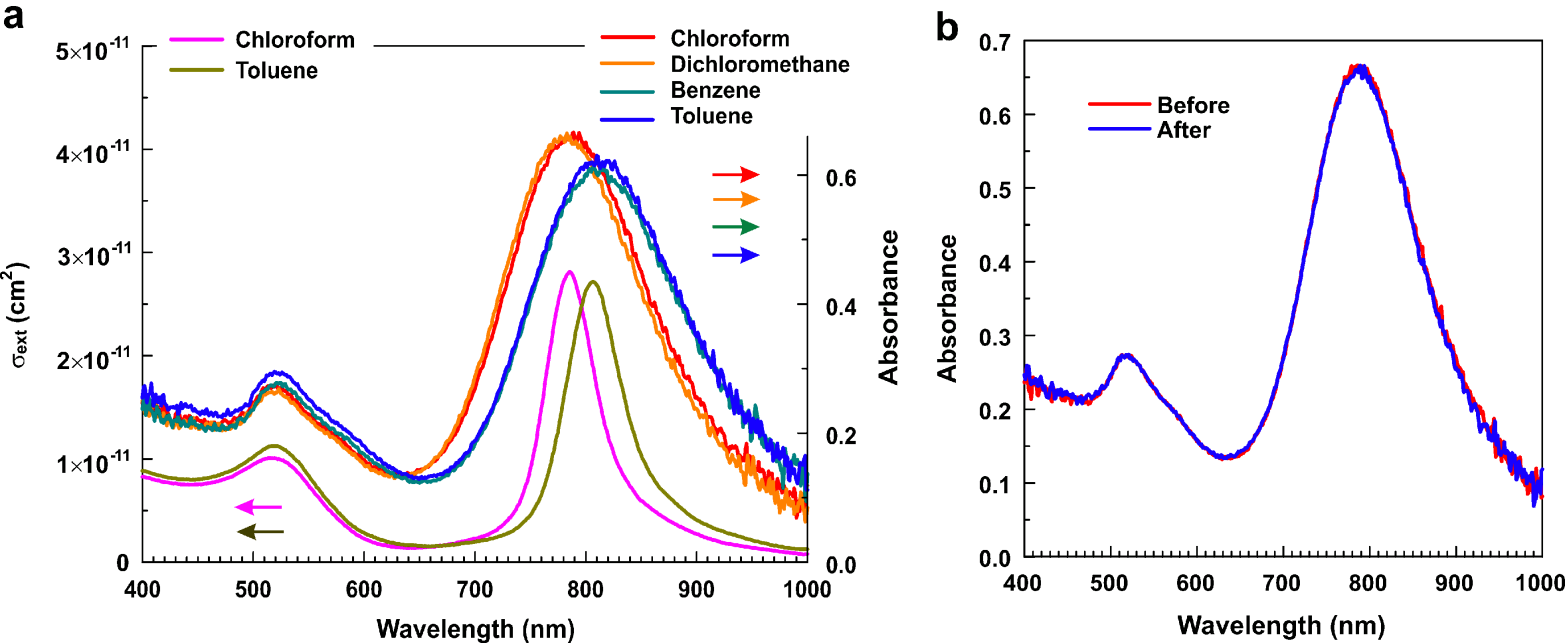
(a) Comparison of the calculated extinction cross sections for the bare single Au nanorod in chloroform and toluene with the optical absorbance spectra of polymer-coated Au nanorods in chloroform, methylene chloride, benzene and toluene. (b) Optical absorbance spectra of polymer-coated Au nanorods repeatedly recorded in chloroform with three in-between cycles of redistribution in methylene chloride, benzene and toluene.

The optical absorbance spectra of Au nanorods measured in different organic solvents demonstrate nearly the same position of the transverse band and pairwise similarity of the longitudinal band between chloroform and methylene chloride, and benzene and toluene, with the latter being red-shifted by ca. 30 nm (Figure 5a). These peculiarities can be caused by polymer-solvent interactions, which are fully reversible. One can also see that the experimental data are in good agreement with the model extinction spectra of Au nanorods in chloroform and toluene. The position of the absorption spectrum maximum appears to be linearly dependent on the refractive index of the dispersion medium (see Figure 3), in accordance with the literature observations [31,45–46].

Figure 5b demonstrates that the absorption spectrum in chloroform remains the same after successive redispersion and drying of Au nanorods in methylene chloride, benzene and toluene.

**Figure 6:**
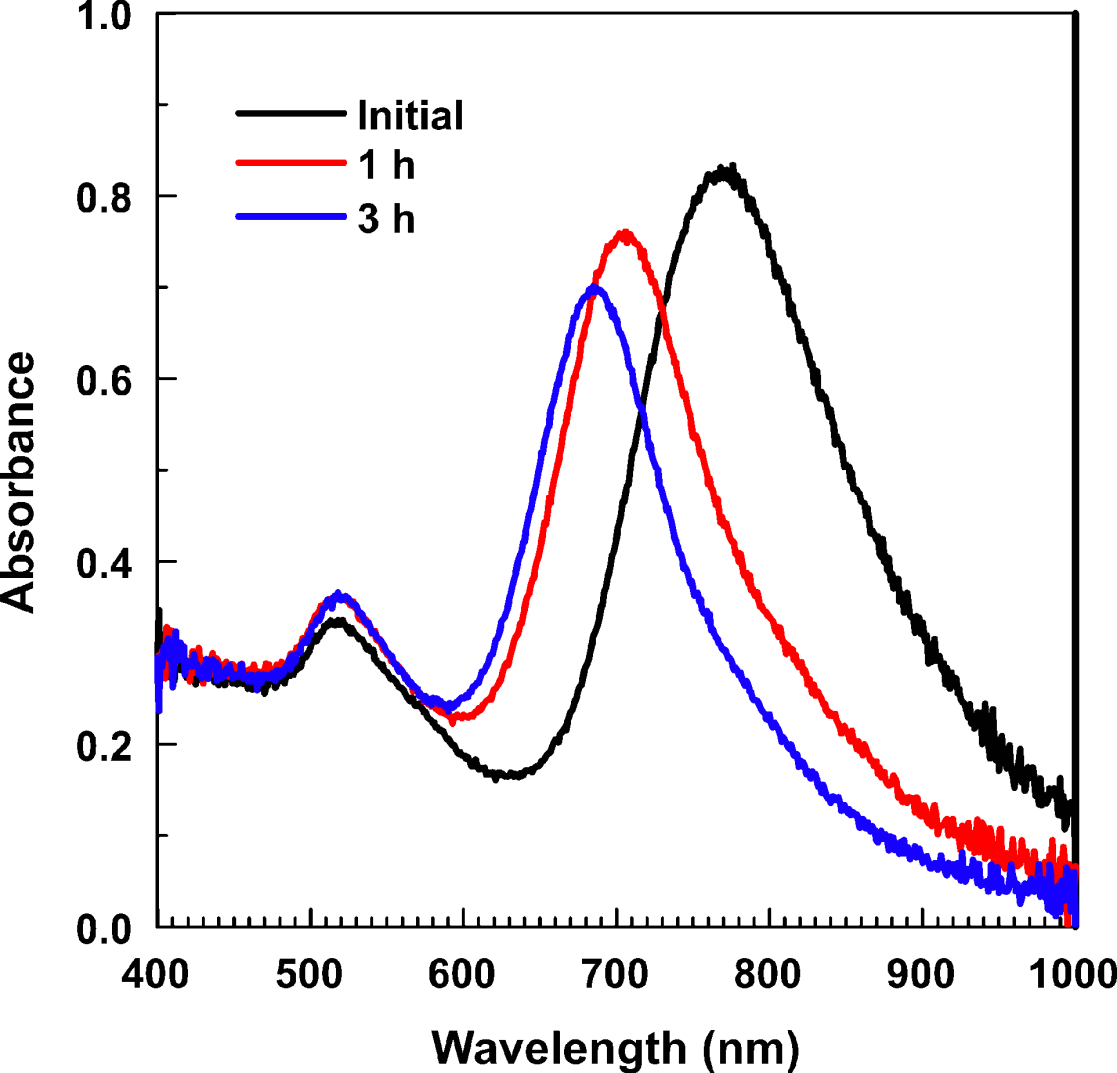
Optical absorbance spectra of polymer-coated Au nanorods in tetrahydrofuran after (black) 0, (red) 1 and (blue) 3 h of aging at 150 °C.

Thermostability is known to be one of the critical issues for gold-containing nanocomposites. As reported in [53], polyvinylpyrrolidone-stabilized Au nanorods of 73 ± 4 nm length and 3.3 ± 0.3 aspect ratio are gradually transformed into spheres in the temperature range of 100–250 °C. At 150 °C, the longitudinal plasmon peak is blue-shifted by 80 nm in 3 h. As seen from Figure 6, in our system, this peak is moved by 85 nm under the same conditions, so that the PS-b-P2VP block copolymer provides similar thermostability to Au nanorods as the polyvinylpyrrolidone homopolymer.

### Stabilization of Au nanospheres

**Figure 7:**
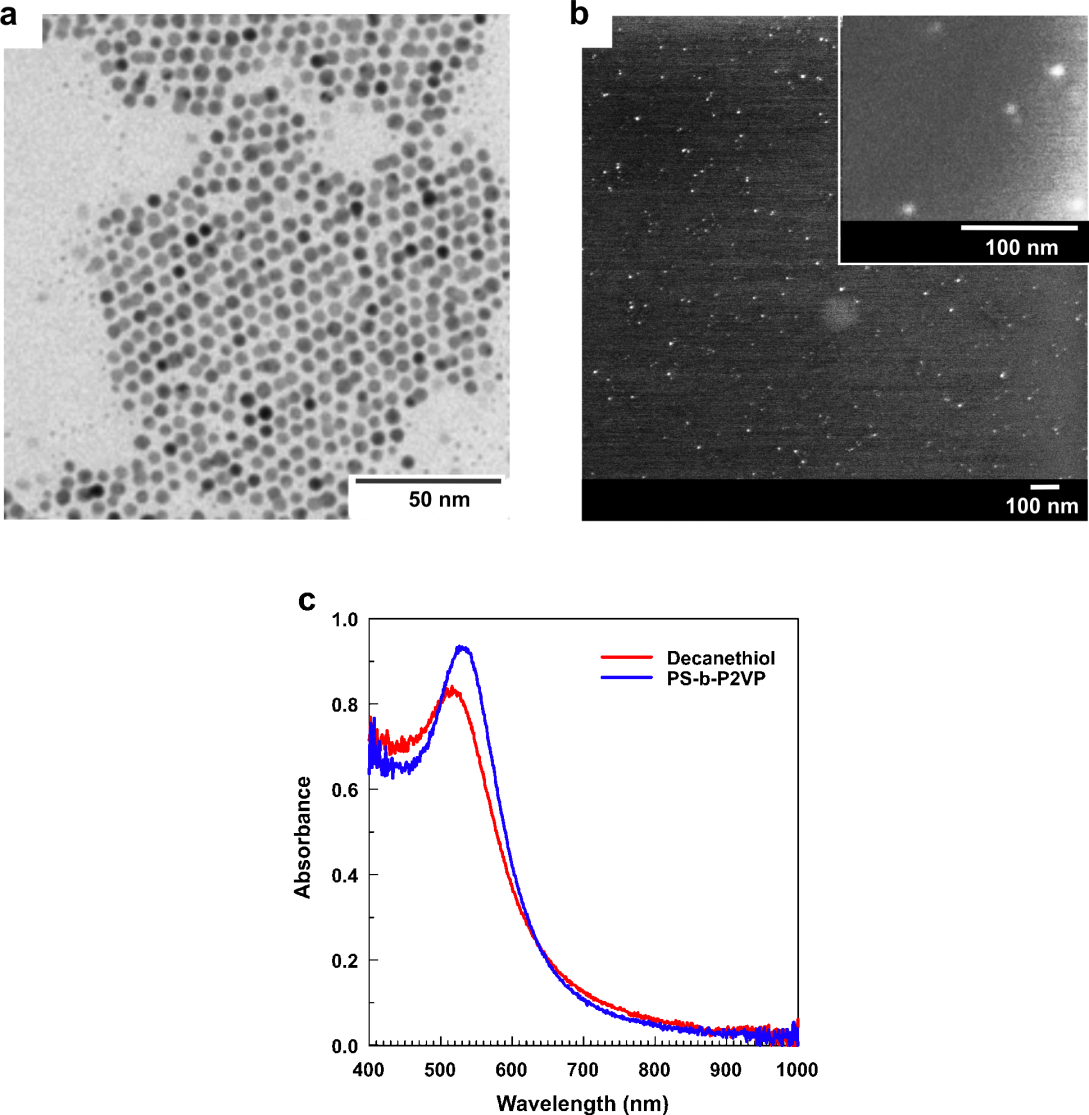
(a) TEM image of decanethiol-stabilized Au nanosphere sol dried from toluene. (b) SEM images of PS-b-P2VP diblock copolymer stabilized Au nanosphere sol dried from toluene. (c) Optical absorbance spectra of Au nanospheres stabilized with (red) decanethiol and (blue) PS-b-P2VP diblock copolymer.

The method of wrapping nanoparticles with diblock copolymers can be also applied to small (5 nm in diameter) organophilic Au nanospheres. In that case, PS-b-P2VP can be introduced in the course of the nanoparticle synthesis instead of decanethiol (for details of both synthetic procedures, see Experimental). As a result, decanethiol- and polymer-coated nanoparticle sols are characterized by qualitatively similar absorption spectra in toluene (Figure 7), whereas polymer-coated nanoparticles reveal considerably higher thermostability.

**Figure 8:**
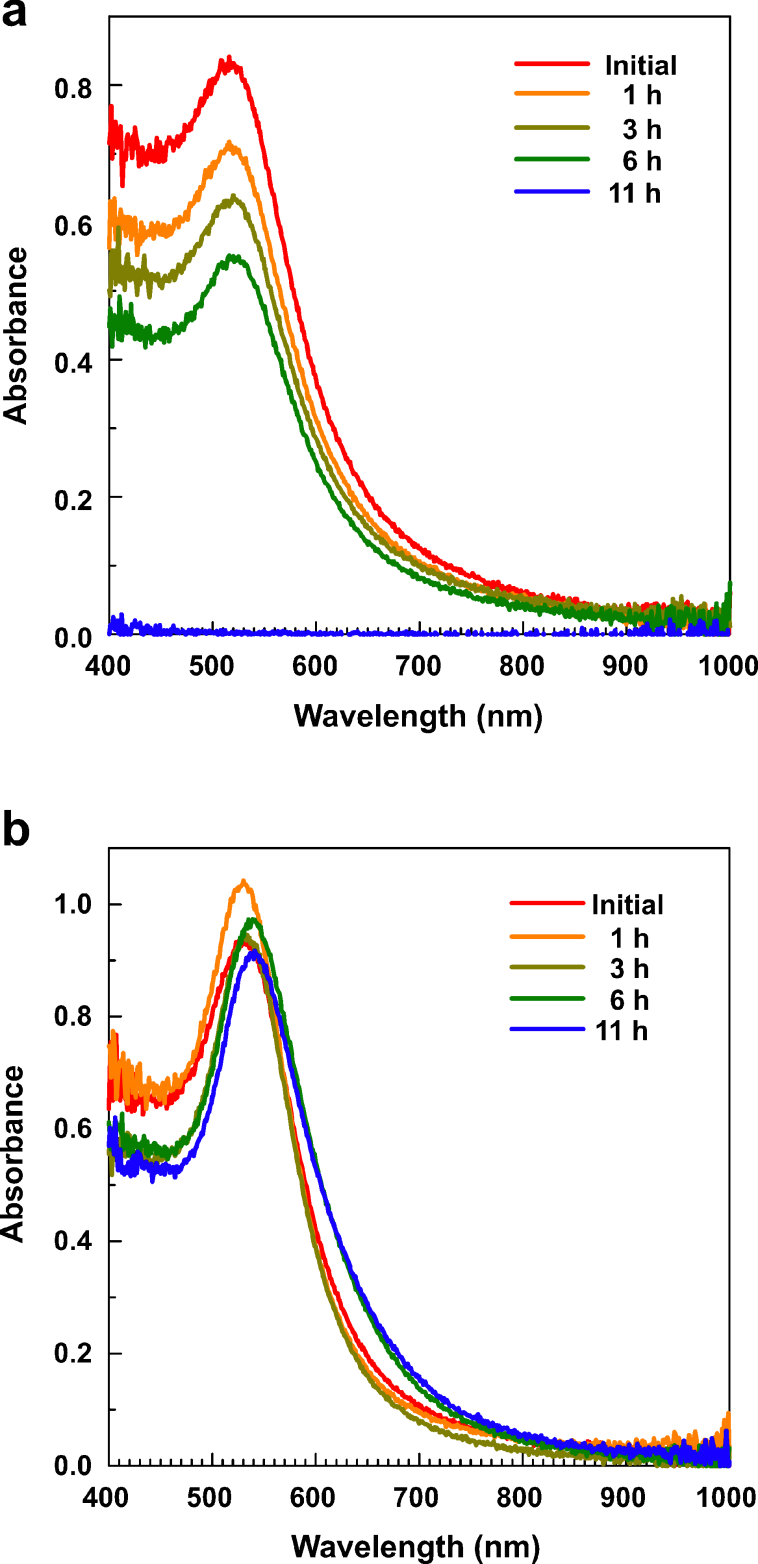
Optical absorbance spectra of (a) decanethiol- and (b) polymer-coated Au nanospheres in toluene after (red) 0, (orange) 1, (olive) 3, (green) 6 and (blue) 11 h of aging at 150 °C.

Indeed, Figure 8 demonstrates that at 150 °C, decanethiol-stabilized Au nanoparticles start to aggregate immediately, and after 11 h of aging, no plasmon resonance is detected. Simultaneously, polymer-stabilized particles keep their optical properties almost unchanged throughout all 11 h of aging.

## Conclusion

In this study, we have shown that CTAB-coated Au nanorods of 30–40 nm length can be rapidly transferred from water to various organic solvents by short-term (5 min) mixing of their hydrosol with a solution of PS-b-P2VP diblock copolymer in tetrahydrofuran. The same copolymer can be used for the stabilization of Au spherical particles of 5 nm diameter. We propose that P2VP blocks wrap around nanoparticles preventing their aggregation, while PS blocks form an organophilic corona that provides dispersability of the nanoparticles in organic media. Compared to thiol- and amine-functionalized stabilizers that form strong covalent bonds with Au, the interaction of pyridine groups with the nanoparticle surface is weaker, thus retaining an opportunity for further changes of the stabilizing ligands. The relative shortcoming of our method is its low net yield, which is below 50% when taking into account both the synthetic and phase transfer stages.

Using TEM and optical absorbance spectroscopy, we have shown that upon phase transfer, the nanoparticles maintain their size almost and do not aggregate in solvents such as chloroform, methylene chloride, tetrahydrofuran, benzene and toluene, even after multiple cycles of drying and redispersion. The thermostability of the nanoparticles is better than in the case of decanethiol stabilization and comparable to what can be achieved with polyvinylpyrrolidone.

We hope that our approach can be a natural route to hybrid composite films. In the microphase-separated state, the copolymer used in this study forms P2VP cylinders of ca. 100 nm diameter in PS bulk, so that Au nanorods of 30–40 nm diameter can be selectively located within the P2VP domains. It is also important that we avoid using an additional third component stabilizer that is usually present in diblock copolymer composites with Au nanoparticles.

## Experimental

### Materials

Tetrachloroauric(III) acid trihydrate (HAuCl_4_·3H_2_O, 99%) was supplied by Aurat, Moscow, Russia. Poly(styrene-b-2-vinylpyridine) (PS-b-2VP, *M*_n PS_ = 380 kg/mol, *M*_n_*_ P_*_2VP_ = 156 kg/mol, *Ð* = 1.23) was purchased from Polymer Source, Canada. All other reagents and solvents (99%) used for nanoparticle synthesis and characterization were obtained from Sigma-Aldrich and used as received or purified according to standard procedures. All the glassware was cleaned by aqua regia and rinsed with distilled water prior to the experiments.

### Nanorod synthesis

Au nanorods were synthesized according to the seeded growth method [14–16] with some modifications. All solutions were prepared with distilled water. In the first stage, a solution of HAuCl_4_·3H_2_O (0.1 mL, 0.01 М) was added to a solution of cetyltrimethylammonium bromide (CTAB, 3.0 mL, 0.1 М) under stirring. When the reaction medium became bright brown-yellow, an ice-water solution of sodium borohydride (NaBH_4_, 0.24 mL, 0.01 M) was added quickly in one portion with vigorous stirring. The seeding time was fixed at 5 min. In the second stage, a growth solution was prepared. Volumes of 7.2 mL of 0.001 М HAuCl_4_ and 0.72 mL of 0.004 М AgNO_3_ solutions were mixed with 14.4 mL of a 0.2 М CTAB solution, followed by adding 0.15 mL of a 0.08 М ascorbic acid solution, with thorough stirring until the medium turned colorless. At this point, 78 μL of the seed solution was introduced and, upon short-term mixing, the growth solution was left for 3 h at 30 °С at rest. Finally, an absorption spectrum was recorded and the resulting ruby-red medium was chilled to 1–5 °С. In the course of cooling, CTAB was partially precipitated and centrifuged at a rate of 6000 rpm for 3 min. The remaining nanoparticle hydrosol was purified and stored in a fridge (yield: 70–90%, fraction of nanorods ≥90%).

### Nanorod organophilization with diblock copolymers

A 3 mL volume of Au nanorod sol was heated to 30 °С and centrifuged at a rate of 15000 rpm for 8 min. The precipitant was dissolved in 1 mL of water to record an absorption spectrum. Then, it was again centrifuged at 15000 rpm for 7 min. The supernatant was discarded and the precipitant was redispersed in 20 µL of water. The nanorod sol, thus concentrated, was added to a 1 mL PS-b-P2VP fresh solution in tetrahydrofuran (3 mg/mL) with a microsyringe at a rate of 1 µL/s under vigorous stirring. During this process, the solution gradually turned the same ruby-red color as the initial hydrosol. After 5 min of stirring, the absorption spectrum was recorded and the solution was rapidly evaporated under vacuum. The residue was dried until constant mass, dissolved in 50 µL of tetrahydrofuran and precipitated by adding 3 mL of ethanol to remove traces of the stabilizer. Then, it was centrifuged at 6000 rpm for 30 min, the precipitant was separated and dried until constant mass (yield 50%). After this procedure, the nanorods can be redispersed in the desired solvent (tetrahydrofuran, chloroform, methylene chloride, toluene or benzene).

### Synthesis of decanethiol-stabilized nanospheres

Spherical Au nanoparticles were synthesized following the method of [54]. An aqueous solution of HAuCl_4_·3H_2_O (0.066 mol per 2.2 mL) was mixed with a solution of tetraoctylammonium bromide (TOAB) in toluene (1.32 mmol per 36.7 mL). After 30 min of vigorous stirring, Au completely transferred into the organic phase, after which the aqueous phase was discarded. An aqueous solution of NaBH_4_ (0.733 mmol per 1.8 mL) was added with a pipette at a rate of one drop (ca. 30 µL) every 15 s under intensive mixing. Upon adding ca. 0.6 mL of NaBH_4_, the organic medium turned colorless, while the next drop made it light brown. After this, all the remaining reducing agent solution was rapidly introduced to the reaction medium, which was stirred for 24 h. A volume of 0.612 mmol of decanethiol was then added followed by 1 h of stirring and solution evaporation to ca. 2 mL, which was stored in a fridge overnight. The next day, the nanoparticles were subjected to precipitation with ethanol, 5 min of centrifugation at 2000 rpm and drying. The precipitation-centrifugation cycle was repeated and the nanoparticles were redispersed in toluene (yield 70%).

### Synthesis of copolymer-stabilized nanospheres

The same method of [54] described above was used before introducing the stabilizer. Instead of decanethiol, a PS-b-P2VP solution in toluene was added and the mixture was heated to 40 °C. The polymer was taken in a 19-fold excess by mass with respect to Au nanoparticles. The medium was stirred for 3 h at 40 °С, then evaporated to dryness, dispersed in a minimum amount of chloroform, precipitated with a 15-fold excess of ethanol, and centrifuged for 20 min at 6000 rpm. After repeating the precipitation-centrifugation cycle, the nanoparticles were redispersed in chloroform (yield 95%).

### Nanoparticle stability tests

Copolymer-stabilized nanorods were dispersed in 1 mL of chloroform to record an absorption spectrum. Then, the solution was evaporated to constant mass under vacuum. This cycle was repeated tenfold and every time an absorption spectrum of the sol was taken.

Copolymer-stabilized nanorods were dispersed in a 1 mL of tetrahydrofuran to record an absorption spectrum. Next, the solution was evaporated to constant mass under a vacuum and aged in an inert atmosphere at 150 °C for 1 h. The procedure was repeated with a longer (2 h) aging, followed by redispersion in 1 mL of tetrahydrofuran and taking an absorption spectrum.

Decanethiol-stabilized nanospheres were dispersed in 1 mL of toluene to record an absorption spectrum. Then, the solution was evaporated to constant mass under a vacuum and aged in an inert atmosphere at 150 °C for 1 h. The procedure was repeated with longer (2, 3 and 5 h) aging and followed by redispersing in 1 mL of toluene and taking an absorption spectrum.

Copolymer-stabilized nanospheres were dispersed in 1 mL of chloroform to record an absorption spectrum. Then, the solution was evaporated to constant mass under a vacuum and aged in an inert atmosphere at 150 °C for 1 h. The procedure was repeated with longer (2, 3 and 5 h) aging, followed by redispersing in 1 mL of chloroform and taking an absorption spectrum.

### Microscopy

TEM images and small area electron diffraction (SAED) patterns were obtained on a transmission electron microscope LEO912 AB OMEGA (Carl Zeiss, Germany) operating at 100 kV. Samples were prepared by drying the sol droplets placed on copper grids coated by Formvar™ film. SAED patterns were obtained at accelerated voltage of 100 kV and drawtube length of 290 mm. Both SAED patterns and dark field images were used to confirm the crystallinity of Au nanoparticles. The length and diameter distributions of the nanorods and nanospheres were measured by ImageJ 1.50b open source software (Wayne Rasband, NIH, USA). SEM images were obtained on a scanning electron microscope JSM 7401F (JEOL, Japan).

### Optical analysis

Absorption spectra of nanoparticle sols in the visible light range were recorded on an optical absorbance USB 2000 spectrometer (Ocean Optics, USA) equipped with a LS-1 halogen light source and a cuvette holder. The spectral data were processed using a SpectraSuite software package (Ocean Optics). Pure solvent absorption spectra were used for comparison.

Simulation of the optical properties was performed with the finite-difference time-domain (FDTD) numerical techniques for Maxwell’s equations solution. Commercially available software FDTD Solutions (Lumerical, Canada) with an evaluation 30-day license was used.
